# Bringing Simulation-Based Education in Psychiatry Into the Virtual Sphere

**DOI:** 10.1192/bjo.2024.325

**Published:** 2024-08-01

**Authors:** Thomas Scurr, Abdallah Adbelkerim, Jessica Scott, Stephen Haupt

**Affiliations:** 1 Devon Partnership Trust, Exeter, United Kingdom; 2 Devon Partnership Trust, Barnstaple, United Kingdom; 3 Livewell Southwest, Plymouth, United Kingdom

## Abstract

**Aims:**

Simulation-based education (SBE) is widespread in both undergraduate and postgraduate medical education, but less frequently in psychiatry. Despite this, the relatively small evidence base suggests high levels of participant satisfaction and educational benefit from SBE in psychiatry. Bringing SBE into the virtual environment presents another set of challenges we identified both through current medical education research and through our own experience. Our poster will demonstrate our current model of virtual simulation, the evidence base we used to develop this, and the feedback we have had from this new venture.

**Methods:**

Background – As part of our undergraduate CAMHS teaching, where students spend 1 week within our service as part of a 3-week psychiatry clinical placement, we provide a single session of CAMHS SBE. This is delivered by 2 facilitators and a professional medical actor providing the role of the adolescent patient. Our virtual simulation teaching session has now been integrated into our teaching program. We have developed this session in line with current medical education research, and have presented this at the Annual Medical Education Conference and integrated feedback on our session into the current model.

**Results:**

We have successfully adapted this session to be delivered remotely, and have received overwhelmingly positive feedback from our students, citing improvements in their confidence and learning after our session. Along with the challenges to engagement, participation, and patient involvement of remote teaching, we further adapted our session to accommodate increased numbers of students attending – a national trend. However, from current research and our experience, there are also benefits to both educators and students from virtual SBE.

**Conclusion:**

Our results show that simulation can be used effectively in psychiatry through virtual media to expand student clinical experience and provide excellent educational opportunities. We present our model for virtual SBE and the evidence base we have used to develop this session, along with the feedback we have had from students, staff, and teams across the country.

